# Exploring the intratumoral heterogeneity of DNA ploidy in prostate cancer

**DOI:** 10.1002/cnr2.1953

**Published:** 2023-12-26

**Authors:** Tatjana Vlajnic, David C. Müller, Christian Ruiz, René Schönegg, Hans‐Helge Seifert, George N. Thalmann, Tobias Zellweger, Clémentine Le Magnen, Cyrill A. Rentsch, Lukas Bubendorf

**Affiliations:** ^1^ Institute of Medical Genetics and Pathology University Hospital Basel, University of Basel Basel Switzerland; ^2^ Department of Urology University Hospital Basel, University of Basel Basel Switzerland; ^3^ Institute of Pathology, Cantonal Hospital St. Gallen St. Gallen Switzerland; ^4^ Department of Urology, Inselspital University Hospital Bern Bern Switzerland; ^5^ Division of Urology St. Clara Hospital Basel Switzerland; ^6^ Department of Biomedicine University Hospital Basel, University of Basel Basel Switzerland; ^7^ Present address: Vancouver Prostate Centre, Department of Urologic Sciences University of British Columbia Vancouver British Columbia Canada

**Keywords:** clonal evolution, heterogeneity, ploidy, prostate cancer

## Abstract

**Background:**

Prostate cancer is morphologically and molecularly heterogeneous. Genomic heterogeneity might be mirrored by variability in DNA ploidy. Aneuploidy is a hallmark of genomic instability and associated with tumor aggressiveness. Little attention has been paid to the biological significance of the diploid tumor cell population that often coexists with aneuploid populations. Here, we investigated the role of DNA ploidy in tumor heterogeneity and clonal evolution.

**Methods:**

Three radical prostatectomy specimens with intratumoral heterogeneity based on nuclear features on H&E were selected. DNA content of each subpopulation was determined by DNA image cytometry and silver in situ hybridization (SISH). Genomic evolution was inferred from array comparative genomic hybridization (aCGH). Additionally, immunohistochemistry was used to examine the stemness‐associated marker ALDH1A1.

**Results:**

Nuclear morphology reliably predicted DNA ploidy status in all three cases. In one case, aCGH analysis revealed several shared deletions and one amplification in both the diploid and the aneuploid population, suggesting that these populations could be related. In the other two cases, a statement about relatedness was not possible. Furthermore, ALDH1A1 was expressed in 2/3 cases and exclusively observed in their diploid populations.

**Conclusions:**

In this proof‐of‐concept study, we demonstrate the feasibility to predict the DNA ploidy status of distinct populations within one tumor by H&E morphology. Future studies are needed to further investigate the clonal relationship between the diploid and the aneuploid subpopulation and test the hypothesis that the aneuploid population is derived from the diploid one. Finally, our analyses pointed to an enrichment of the stemness‐associated marker ALDH1A1 in diploid populations, which warrants further investigation in future studies.

## 1 INTRODUCTION

Prostate cancer (PC) is morphologically and molecularly heterogeneous. One way to measure tumor heterogeneity is at the level of DNA ploidy. Aneuploidy is a hallmark of genomic instability and associated with tumorigenesis^1^ and adverse outcome in PC.^2^, ^3^, ^4^, ^5^, ^6^, ^7^, ^8^ Moreover, assessment of DNA ploidy status by machine learning‐based image cytometry^9^, ^10^ was proposed as an adjunct for the risk stratification of PC.

It has also been recognized that diploid and aneuploid tumor cells commonly coexist in PC.^5^, ^11^, ^12^ However, the biological significance of intratumoral heterogeneity of the ploidy status and the question of whether the diploid and aneuploid subpopulations within a tumor are related to each other remains to be clarified.

Here, we aimed to predict the ploidy status of distinct populations within one tumor area by hematoxylin and eosin (H&E) morphology and to investigate the clonal relationship between diploid and aneuploid tumor cell populations.

## 2 MATERIALS AND METHODS

### 2.1 Study population/Tissue samples

Radical prostatectomy specimens with prostate adenocarcinoma were retrieved from the archives of the Institute of Pathology Basel. We specifically selected cases with striking morphological discrimination between a diploid‐ and an aneuploid‐looking cell subpopulation, based on the nuclear features on H&E, irrespective of Gleason pattern. Additionally, these distinct populations needed to be adjacent to each other or within the same tumor area. We defined morphological criteria for the distinction between diploid‐ and aneuploid‐like cells as follows: “diploid‐like” tumor cells: monomorphic nuclei (small, round) with small and inconspicuous nucleoli, fine and evenly distributed chromatin, and a low nuclear/cytoplasmic ratio; “aneuploid‐like” tumor cells: nuclear pleomorphism and large nuclei (≥2× the diameter of a benign glandular prostate cell), large nucleoli, irregular and coarse chromatin, and a high nuclear/cytoplasmic ratio (Table S1). This study was approved by the Ethics Committee of Northwestern and Central Switzerland (EKNZ, No. EK13/11).

### 2.2 Enrichment for tumor cells

To enrich for tumor cells in the two morphologically identified distinct tumor regions, we dissected the respective areas of the original formalin‐fixed paraffin‐embedded (FFPE) blocks and re‐embedded them in separate FFPE blocks.

### 2.3 Silver in situ hybridization

Tissue sections of 4 μm were cut from FFPE blocks. Silver in situ hybridization (SISH) with centromere probes for chromosomes (CEP) 7, 17, and 18 was performed according to the manufacturer's protocols using the Ventana ultraView SISH Detection Kit on the BenchMark XT automated slide stainer (Ventana Medical System Inc). Fifty tumor cells were scored for each probe, and the mean value was determined, respectively. Diploid status was defined as mean values between 1.5 and 2 signals per CEP and tetraploid/aneuploid status as mean values of >2 signals per CEP. Of note, it cannot be excluded that some cells with values around 4 signals represent diploid tumor cells in a G2 phase of the cell cycle.

### 2.4 DNA image cytometry

DNA image cytometry (DNA ICM) was performed at the Institute of Pathology, Cantonal Hospital St. Gallen. Between 262 and 453 cells were selected from each slide for manual DNA measurement by static DNA ICM using an AutoCyte QUIC DNA workstation (TriPath Imaging Inc.) on H&E prestained and Feulgen‐restained slides. Benign fibroblasts served as reference cells for a diploid DNA content (2c). DNA ploidy classes were defined as (i) diploid with a main peak in the 2c region, (ii) tetraploid with a main peak in the 4c region, and (iii) aneuploid with a main peak around 4c region and a varying number of cells outside. Of note, it cannot be excluded that some cells with a peak around 4c region represent diploid tumor cells in a G2 phase of the cell cycle.

### 2.5 Immunohistochemistry

Tissue sections of 4 μm were cut from FFPE blocks. Standard indirect immunoperoxidase procedures were used for the detection of Ki67 (clone MIB1, prediluted, DAKO) and ALDH1A1 (clone ab51028, Abcam) at 1:200 dilution as previously reported.^13^ The analyses were performed on the BenchMark XT automated immunostainer using the OptiView detection system (Ventana Medical System Inc.).

### 2.6 Array comparative genomic hybridization

DNA extraction was performed with the Maxwell 16 Tissue DNA Purification Kit (Promega Corporation). We used an input of 100 ng tumor DNA per sample and performed aCGH, as previously described.^14^

## 3 RESULTS

### 3.1 Correlation between nuclear morphology and DNA ploidy

**FIGURE 1:**
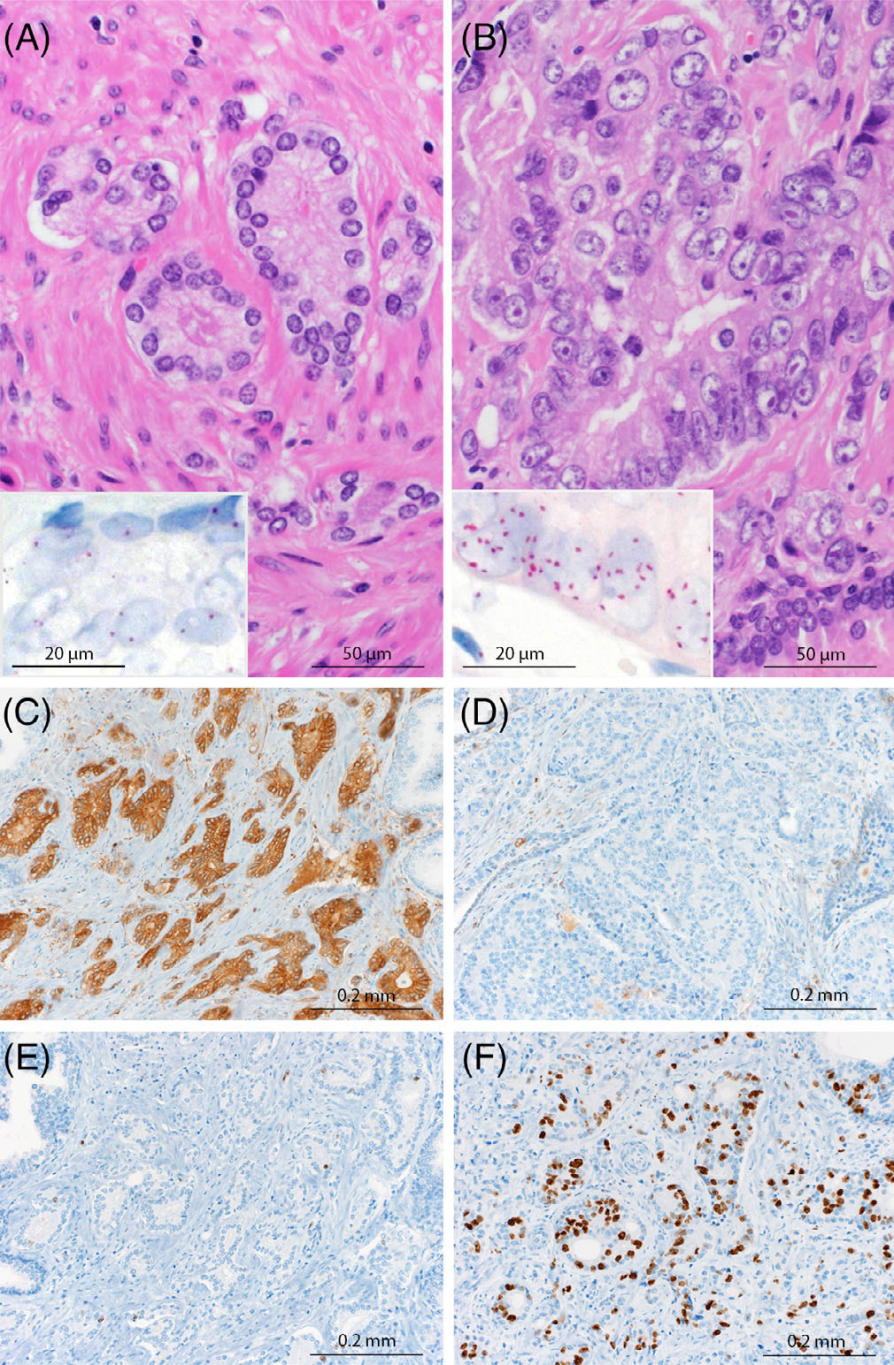
Case 1. (A, B) Morphology of the diploid‐like (A) and aneuploid‐like (B) tumor populations (H&E, magnification 200×). Note that the diploid‐like population (A) consists of monomorphic tumor cells with small round nuclei with inconspicuous nucleoli whereas the aneuploid‐like population (B) shows anisonucleosis and pleomorphic large nuclei with prominent nucleoli. Inset: Tumor cells with a normal (1–2 signals per nucleus) copy number (A) and increased (8–10 signals per nucleus) copy number (B) of CEP17 signals (SISH, magnification 400×). (C, D) Diffuse cytoplasmic positivity for ALDH1A1 in the diploid (C) and complete negativity in the aneuploid (D) tumor population (scattered basal cells in normal glands as positive internal control). (E, F) Low proliferation index (Ki67 LI) in the diploid (E) and markedly higher Ki67 LI in the aneuploid (F) tumor population (C–F magnification 200×).

The DNA content of each subpopulation was estimated by SISH and DNA ICM. In all three cases, analysis of nuclear morphology predicted DNA ploidy defined by SISH (Figure 1A,B). DNA ICM was not available for one patient (case 2; Figures S1 and S2). For the other two, DNA ICM was consistent with the ploidy status as defined by morphology and SISH (Table 1).

**TABLE 1:** Summary of the results.

Case	H&E	DNA ICM (mean peak)	SISH (mean for CEP 7, 17, 18)	ALDH1A1	Ki67 LI (%)	ArrayCGH
1a	DL	Diploid (2.08c)	<2 (1.9; 1.7; 1.9)	Positive	5	No shared CNVs
1b	AL	Non‐diploid (3.76c)	>2 (3.7; 3.4; 4.0)	Negative	20	
2a	DL	N.A.	<2 (1.8; 1.8; 1.9)	Positive	5	Shared CNVs
2b	AL	N.A.	>2 (2.9; 2.9; 3.0)	Negative	10	
3a	DL	Diploid (2.02c)	<2 (1.7; 1.5; 1.6)	Negative	5	No shared CNVs
3b	AL	Non‐diploid (3.89c)	>2 (2.9; 2.1; 2.9)	Negative	15–20	

*Abbreviations*: AL, aneuploid‐like; ArrayCGH, array comparative genomic hybridization; CEP, centromere probes; CNVs, copy number variations; DL, diploid‐like; DNA ICM, DNA image cytometry; H&E, hematoxylin and eosin; Ki67 LI, Ki67 labeling index; N.A., not available; SISH, silver in situ hybridization.

### 3.2 Possible relationship between the diploid and the aneuploid tumor population

**FIGURE 2:**
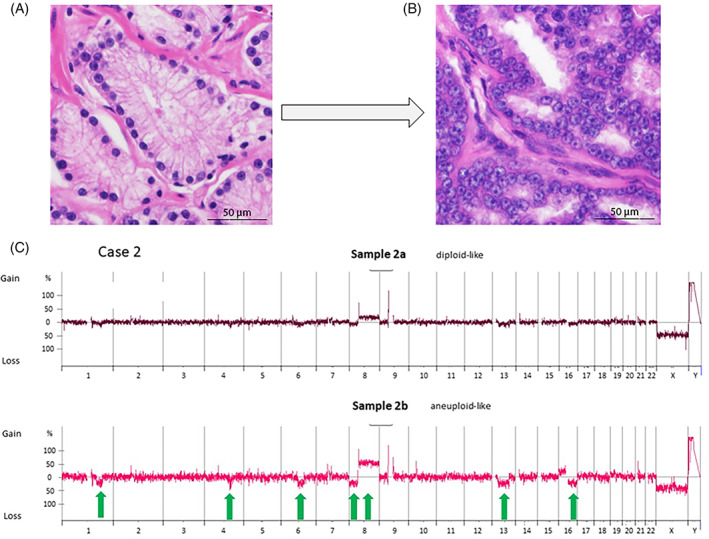
Case 2. (A, B) Morphology of the diploid‐like (A) and aneuploid‐like (B) tumor populations (H&E, magnification 400×). Note that the diploid‐like population (A) has monomorphic tumor cells with small round nuclei and inconspicuous nucleoli whereas the aneuploid‐like population (B) shows larger nuclei with prominent nucleoli and a high nuclear/cytoplasmic ratio. C aCGH‐profiles indicating several shared chromosomal aberrations such as shared deletions (on chromosomes 1q, 4q, 6q, 8p, 13q, and 16q) and one amplification (on chromosome 8q) in both the diploid and the aneuploid populations. Numbers indicate chromosomes. The *y*‐axis indicates copy number changes. Green arrows indicate relevant (shared) copy number changes.

In case 2, we identified several shared deletions (on chromosomes 1q, 4q, 6q, 8p, 13q, and 16q) and one amplification (on chromosome 8q) in both the diploid and the aneuploid population, suggesting that these populations could be related (Figure 2). The aneuploid population additionally showed an amplification on chromosome 16p. While this observation only points to a possible relatedness, this assumption needs to be proven by additional molecular methods.

In cases 1 and 3, aCGH did not reveal any unequivocal copy number variations (CNVs) in the diploid populations, which may partly be explained by a very low tumor cell content. On the contrary, the aneuploid tumor populations showed several CNVs, respectively (Figures S3 and S4) Therefore, any statement about relatedness is not possible for these two cases.

### 3.3 Expression of the stemness‐associated marker in the diploid tumor population

ALDH1A1 was expressed in 2/3 cases and exclusively observed in their diploid population (Figures 1C,D and S5). In all three cases, the fraction of Ki67‐positive tumor cells was lower in the diploid population (5%, respectively) than in the aneuploid population (range: 10–20%; median 17%; Figure 1E,F).

## 4 DISCUSSION

The transition from a diploid state to an aneuploid state over time has been studied in precancerous lesions of the uterine cervix^15^ and Barrett dysplasia in the esophagus.^16^ Likewise, it is generally accepted that in invasive carcinoma, aneuploidy results from chromosomal changes acquired during progression of initially diploid tumors.^17^ Here, we demonstrate the coexistence of adjacent diploid and aneuploid subpopulations within one tumor. Moreover, we show that ploidy defined by DNA ICM and SISH can be predicted by morphology on H&E, based on the degree of nuclear atypia (Figure 1A,B). The robustness of this observation needs to be explored in larger study cohorts. For the moment, it is important to verify the ploidy status using DNA cytometry or suitable molecular methods and not entirely rely on histomorphology. Furthermore, with the methods described, it is not possible to discriminate between a diploid tumor cell in G2 phase of the cell cycle from a tetraploid tumor cell on a single cell basis. However, given that we defined the aneuploid cell population based on evaluation of 50 tumor cells by SISH and > 200 tumor cells by DNA ICM, it seems rather unlikely that all these cells would be in a G2 phase at the same time. Furthermore, the morphological features that we observed in association with the aneuploid cell population, are not expected to occur in diploid cells in a G2 phase.

The heterogeneity of DNA ploidy in PC has been a topic of interest in studies dating from over 20 years ago, focusing on the prognostic value.^5^, ^18^, ^19^ Likewise, Helpap et al. defined morphological criteria for a nuclear grading based on features of nuclei, chromatin, and nucleoli^20^, ^21^ and proposed to combine it with the Gleason grading to improve grading accuracy.^22^, ^23^ However, nuclear features have not become part of the routine diagnostic assessment.^24^

More recently, Andor et al.^25^ found that higher morphological heterogeneity, defined by variability in nuclear size and hyperchromasia, was associated with higher genetic heterogeneity across different tumor types. In light of this evidence, we emphasize the importance of nuclear morphology as a surrogate marker of DNA ploidy and genomic instability with potential prognostic impact.

Exploring the clonal relationship between diploid and aneuploid populations by aCGH in our study was challenged by the notoriously low number of CNVs in diploid populations of early PCs. Whole exome sequencing would be a more sensitive approach to study clonal relationship, yet the feasibility of this approach was limited by the small specimen size of the diploid‐like tumor populations and formalin fixation. As opposed to previous studies investigating tumor heterogeneity in separate tumors within the prostate with regard to the origin of multifocal disease,^11^, ^18^ we examined the relationship between the diploid and aneuploid population within the same tumor area, which, to the best of our knowledge, has not been addressed before. Following the assumption that the aneuploid population is derived from the diploid one, we expected to find shared chromosomal aberrations within the two populations as well as additional aberrations in the aneuploid population that were acquired over time. Indeed, all three diploid populations showed a virtually flat copy number profile, whereas numerous aberrations were present in the aneuploid populations. In case 2, aCGH analysis revealed a subset of shared chromosomal aberrations in both populations, which may point to a possible relatedness (Figure 2). However, these data are not sufficient to draw any conclusion regarding clonal relationship, which needs to be investigated with additional molecular methods. In the other two patients, aCGH did not allow for reliable comparison of the two populations either due to lack of CNVs or insufficient content of tumor DNA of the diploid populations. Interestingly, the non‐diploid populations had a near‐tetraploid stemline, suggesting that the diploid population had undergone whole‐genome doubling (WGD) during evolution. WGD has recently been recognized as an important and recurrent event during the progression of solid tumors.^26^

We have previously investigated longitudinal clonal evolution over an 8‐year period on a multiple biopsy series from a single patient with PC.^14^ Similar to the present study, a diploid and an aneuploid population were identified at the various time points showing several shared and unique genomic aberrations. Interestingly, a series of identical aberrations was present in the diploid populations across all samples, suggesting that this diploid fraction represented the original clone and served as a backbone of clonal evolution. A similar study examined clonal evolution over time in a single patient with metastatic PC.^27^ Interestingly, the clonal population found in the metastases originated from a small and well‐differentiated (Gleason pattern 3) carcinoma focus in the radical prostatectomy specimen and not from the predominant, poorly differentiated component (i.e., Gleason pattern 4). This conclusion was based on the detection of shared gene mutations in the focal Gleason pattern 3 primary tumor and distant metastases, respectively, but not in the predominant Gleason pattern 4 component in the primary tumor. The data from these two published cases indicate that a clone found in the diploid tumor cell population may preferentially drive tumor progression and metastasis. Based on this observation, we hypothesized that the diploid population could be enriched for tumor‐initiating cancer stem cells (CSCs). While the phenotype of prostate CSC is often debated (47), aldehyde dehydrogenase isoform ALDH1A1 represents a promising marker, which has been associated with stemness properties in PC.^13^, ^28^, ^29^ Interestingly, in 2/3 cases, we found a diffuse expression of ALDH1A1 restricted to the diploid population, which was not observed in the aneuploid population (Figures 1C,D and S5). This may suggest an enrichment of specific CSC in diploid populations. However, this hypothesis needs to be investigated on a larger number of samples and preferably with additional CSC markers.

## 5 CONCLUSION

In this proof‐of‐concept study, we showed that it is possible to predict the DNA ploidy status of distinct populations within one tumor by the degree of morphological nuclear atypia by H&E. Further analyses in larger study cohorts and with more sensitive approaches are needed to investigate clonal relationship between the diploid and the aneuploid populations and our assumption that the aneuploid population might arise from a WGD event. Finally, our observation that the stemness‐associated marker ALDH1A1 is restricted to specific diploid populations, warrants further investigations.

## AUTHOR CONTRIBUTIONS

**Tatjana Vlajnic:** Conceptualization (equal); data curation (lead); formal analysis (lead); methodology (equal); writing – original draft (lead). **David C. Müller:** Data curation (equal); formal analysis (equal); writing – original draft (equal). **Christian Ruiz:** Formal analysis (equal); validation (equal). **René Schönegg:** Formal analysis (equal); validation (equal). **Hans‐Helge Seifert:** Resources (equal); writing – review and editing (supporting). **George N. Thalmann:** Resources (supporting); writing – review and editing (supporting). **Tobias Zellweger:** Resources (equal); writing – review and editing (supporting). **Clémentine Le Magnen:** Writing – review and editing (supporting). **Cyrill A. Rentsch:** Resources (lead); writing – review and editing (equal). **Lukas Bubendorf:** Conceptualization (lead); funding acquisition (lead); methodology (lead); project administration (lead); supervision (lead); writing – review and editing (lead).

## FUNDING INFORMATION

None.

## CONFLICT OF INTEREST STATEMENT

The authors declare that they have no conflicts of interest. This work has previously been published as an abstract in Pathologe 2015 · 36:[Suppl 1] · 2–138: FR‐053 Heterogeneity of DNA ploidy as a hallmark of clonal evolution in prostate cancer.

## ETHICS STATEMENT

This study was approved by the Ethics Committee of Northwestern and Central Switzerland (EKNZ, No. EK13/11). Personal informed consent was not required for this study according to the ethical approval.

## Supporting information


Figure S1.
Figure S2.
Figure S3.
Figure S4.
Figure S5.
Table S1.

## Data Availability

I confirm that my article contains a Data Availability Statement even if no data is available (list of sample statements) unless my article type does not require one.
