# Supplementary material for: Exploring the intratumoral heterogeneity of DNA ploidy in prostate cancer
Source: Cancer Rep (Hoboken). 2023 Dec 26;7(2):e1953. doi: 10.1002/cnr2.1953 (PMC10849929; doi:10.1002/cnr2.1953)
Supplement: Supplementary file 1 — Figure S1. Figure S2. Figure S3. Figure S4. Figure S5. Table S1. [file CNR2-7-e1953-s001.docx]

**SUPPLEMENTARY FIGURES**


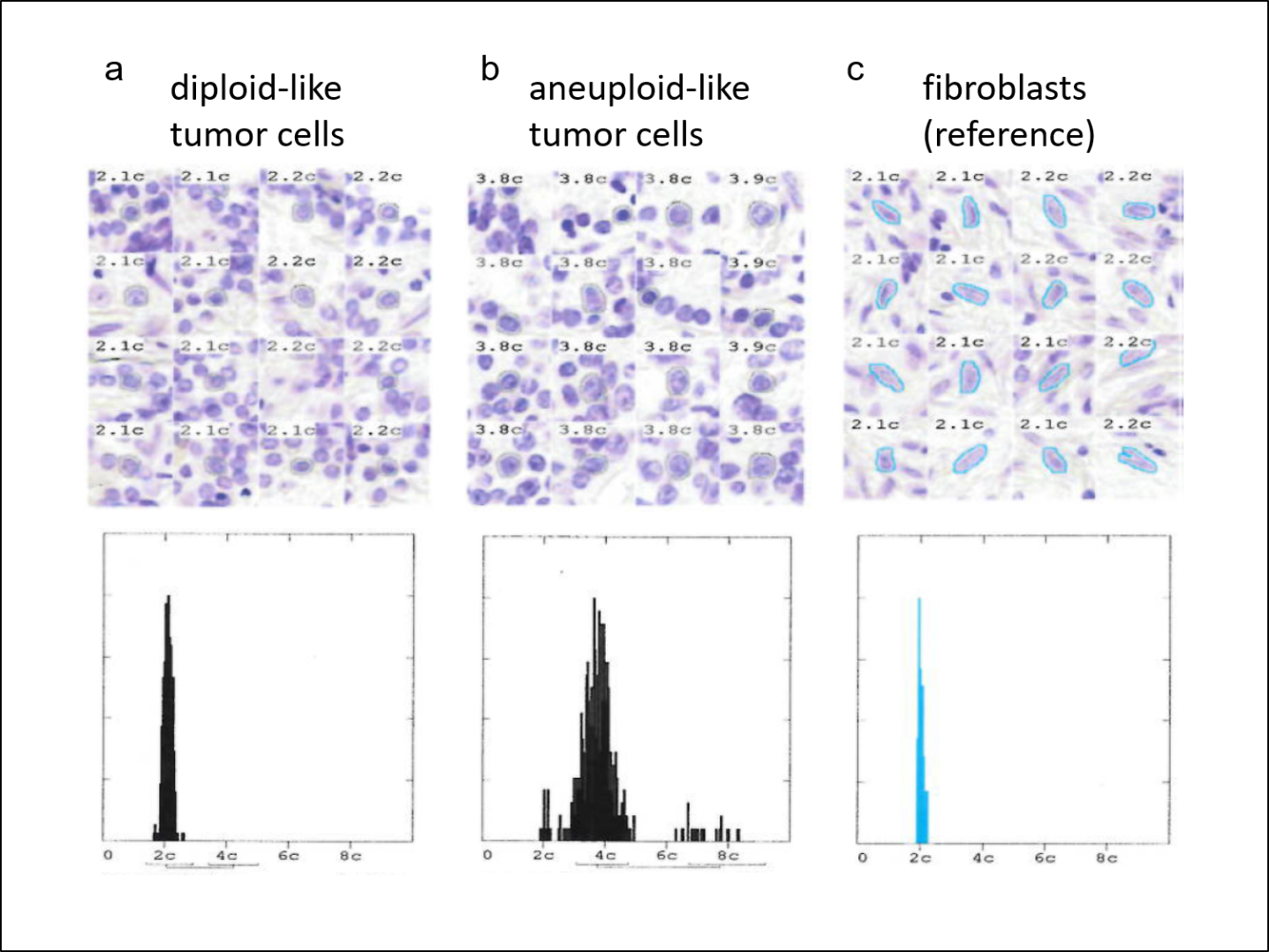


**Suppl. Fig. 1.** DNA image cytometry of case 1 showing a) diploid-like tumor cells with a peak around 2 b) aneuploid-like tumor cells with a peak around 4c c) fibroblasts as reference with a peak around 2c.


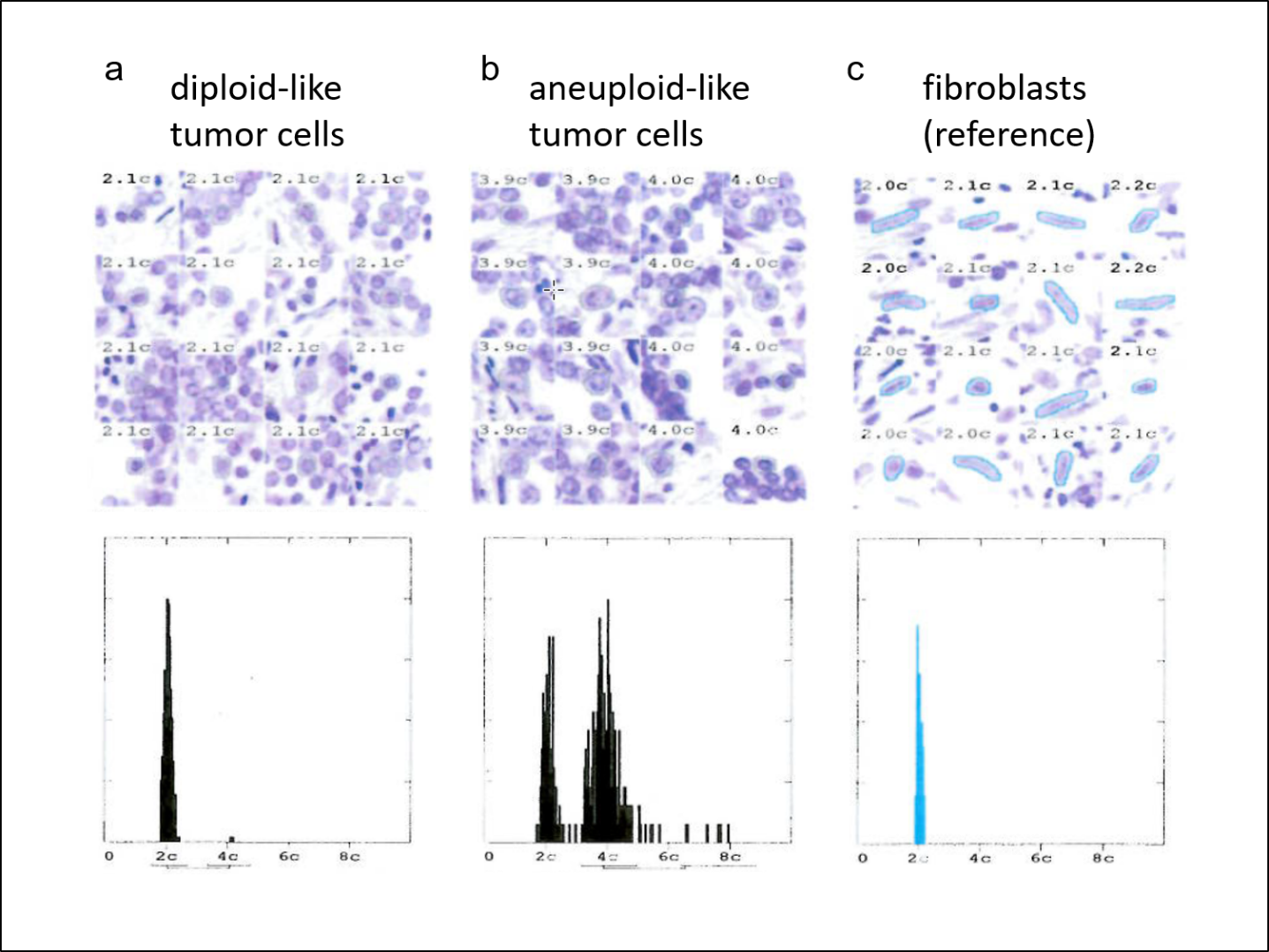


**Suppl. Fig. 2.** DNA image cytometry of case 3 showing a) diploid-like tumor cells with a peak around 2 b) aneuploid-like tumor cells with a peak around 4c c) fibroblasts as reference with a peak around 2c.


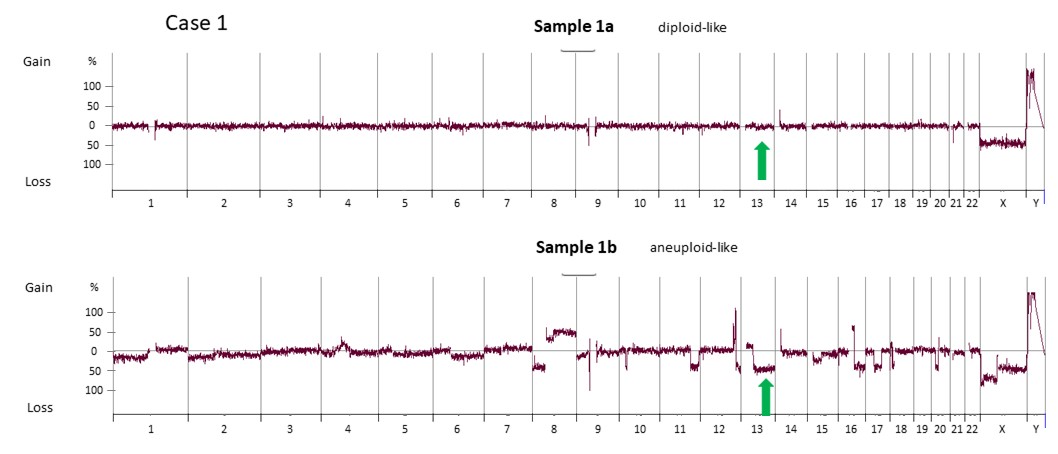


**Suppl. Fig. 3**. Case 1. aCGH-profiles indicating a presumable deletion on chromosome 13q (green arrow) in the diploid tumor population and numerous copy number changes (deletions on chromosomes 8p, 11q, 15p, 16q, 17p, 20p; amplification on 8q), including the deletion on 13q in the aneuploid tumor population (green arrow).


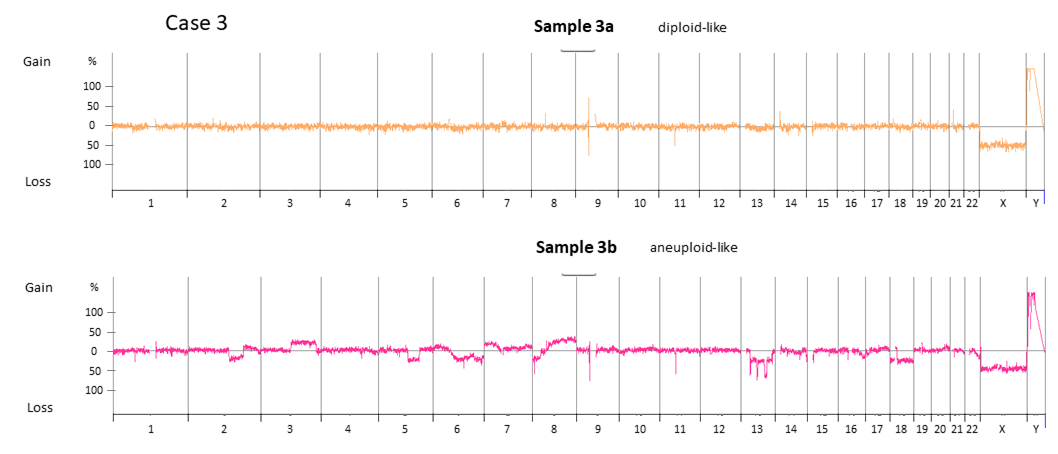


**Suppl. Fig. 4.** Case 3. aCGH-profiles indicating presumable deletions on chromosomes 6q, 13q, and 18q in the diploid tumor population and additional copy number changes, including the deletion on 6q, 13q, and 18q in the aneuploid tumor population.


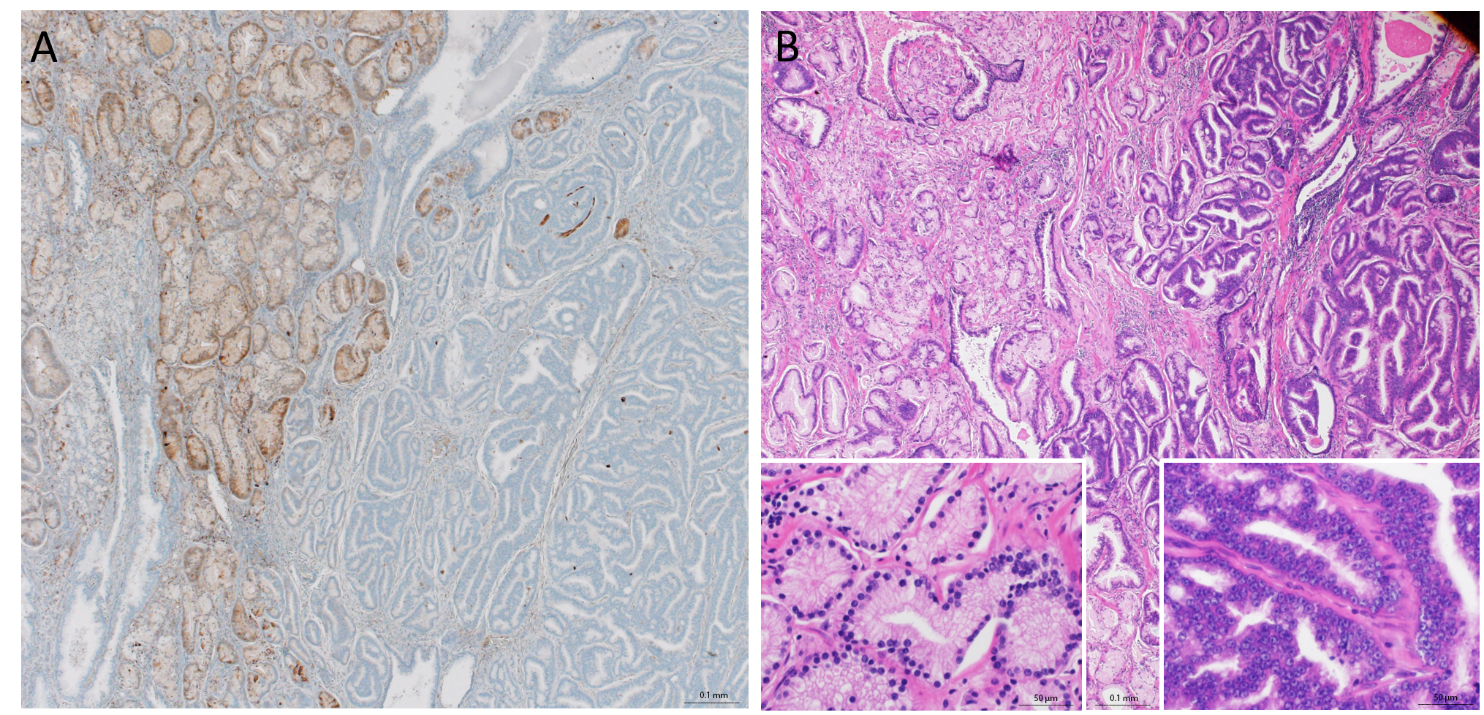


**Suppl. Fig. 5.** Case 2. A Diffuse cytoplasmic positivity for ALDH1A1 in the diploid (left side) and complete negativity in the aneuploid (right side) tumor population. B Morphology of the diploid-like (left side) tumor population with monomorphic tumor cells with small round nuclei and inconspicuous nucleoli whereas the aneuplpoid-like tumor population (right side) shows larger nuclei with prominent nucleoli and a high nuclear/cytoplasmic ratio (H&E, magnification 40x, inset: magnification 400x).

**SUPPLEMENTARY TABLE**

|  | “Diploid-like” tumor cells | “Aneuploid-like” tumor cells |
| --- | --- | --- |
| Nuclei | Monomorphic, small, round, regular membranes | Anisonucleosis, pleomorphic, large*, irregularly shaped, irregular membranes |
| Chromatin | Fine, evenly distributed | Coarse, irregular |
| Nucleoli | Small, inconspicuous | Large, red, several in number |
| N/C Ratio | Low | High |

**Suppl. Table 1**. Morphological criteria for “diploid-like” and “aneuploid-like” tumor cells. N/C, nuclear/cytoplasmic.

* ≥2x the diameter of a benign glandular prostate cell
